# Spontaneous regression of a rectal tonsil presenting as a large submucosal tumor

**DOI:** 10.1002/deo2.34

**Published:** 2021-09-01

**Authors:** Toru Matsui, Eri Naitoh, Kengo Furutani, Tomoji Katoh, Katsuya Kobayashi, Kenichiro Sekigawa, Hiroshi Mitsui

**Affiliations:** ^1^ Department of Gastroenterohepatology Tokyo Teishin Hospital Tokyo Japan

**Keywords:** colonoscopy, lymphoid tissue, polyp, rectum, submucosa

## Abstract

Rectal tonsils are localized hyperplastic lymphoid tissues in the rectum, and the initial endoscopic findings are consistent with those for neoplastic lesions. However, rectal tonsils are benign entities, and the diagnosis should be made cautiously. A 70‐year‐old man presented with pain on defecation with rectal bleeding. Colonoscopy revealed a 3‐cm protruding mass in the rectum with mucosal erosion, but no malignant features were observed on forceps biopsy. Endoscopic ultrasonography (EUS) showed that the lesion was a hypoechoic mass without blood flow. Fine needle aspiration under EUS revealed no malignant components, although the size of the lesion had shrunk, and symptoms, such as blood‐stained stool, tenesmus, and discomfort during defecation, had resolved. A second forceps biopsy showed intermediate‐sized lymphocytes without lymphoepithelial lesions. Based on immunostaining, the lesion was diagnosed as a rectal tonsil.

Rectal tonsils occur due to localized proliferation of reactive lymphoid follicles in the submucosa or muscularis mucosa. However, endoscopic diagnosis is difficult since less invasive treatment is performed for neoplastic lesions of the rectum to preserve the function of the anal sphincter. Diagnosis and treatment of small lesions might be possible by endoscopic resection; however, for relatively large lesions, formulating a diagnosis based only on biopsy specimens becomes even more difficult. Therefore, repeated biopsies might be helpful for the diagnosis of rectal tonsils and for excluding other neoplasms.

## INTRODUCTION

Rectal tonsils (RTs) are localized hyperplastic lymphoid tissues in the rectum, and the initial endoscopic diagnosis is consistent with that for neoplastic lesions.^1^, ^2^ However, RTs are benign entities, and diagnosis should be made cautiously. RTs occur due to localized proliferation of reactive lymphoid follicles in the submucosa or muscularis mucosa. However, endoscopic diagnosis is difficult^3^ since less invasive treatment is performed for neoplastic lesions of the rectum to preserve the function of the anal sphincter. Diagnosis and treatment of small lesions might be possible by endoscopic resection; however, for relatively large lesions, formulating a diagnosis based only on biopsy specimens becomes even more difficult. Therefore, repeated biopsies might be useful for the diagnosis of RTs and for excluding other neoplasms. Herein, we describe a rare case of RT presenting as a large submucosal tumor (SMT) diagnosed by forceps biopsy. The tumor was observed to change its form from an SMT to a scarred lesion.　This study was conducted according to the principle of the Declaration of Helsinki. The patient provided written informed consent, and the study was approved by the appropriate ethics review board.

## CASE REPORT

**Figure 1:**
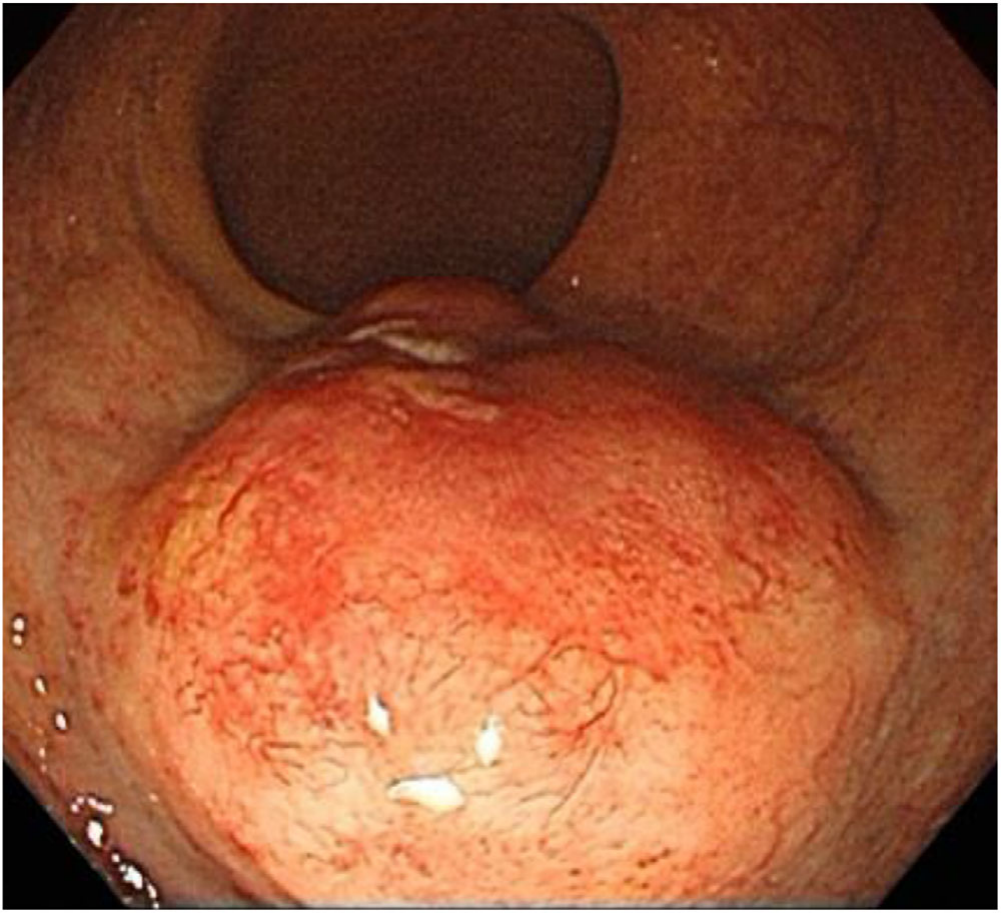
Endoscopy shows a submucosal tumor (diameter, 30 mm) in the lower rectum

**Figure 2:**
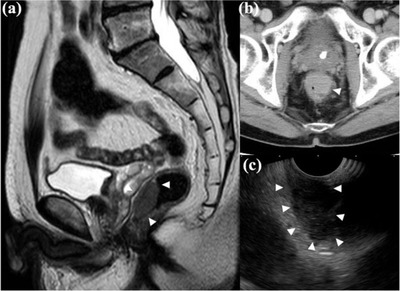
(a) T2‐weighed magnetic resonance imaging showing a well‐defined, elevated lesion in the anterior rectal wall. (b) Contrast‐enhanced computed tomography shows a lower rectal mass with a contrast‐enhancing effect. (c) Endoscopic ultrasonography demonstrates a heterogeneous hypoechoic lesion.

**Figure 3:**
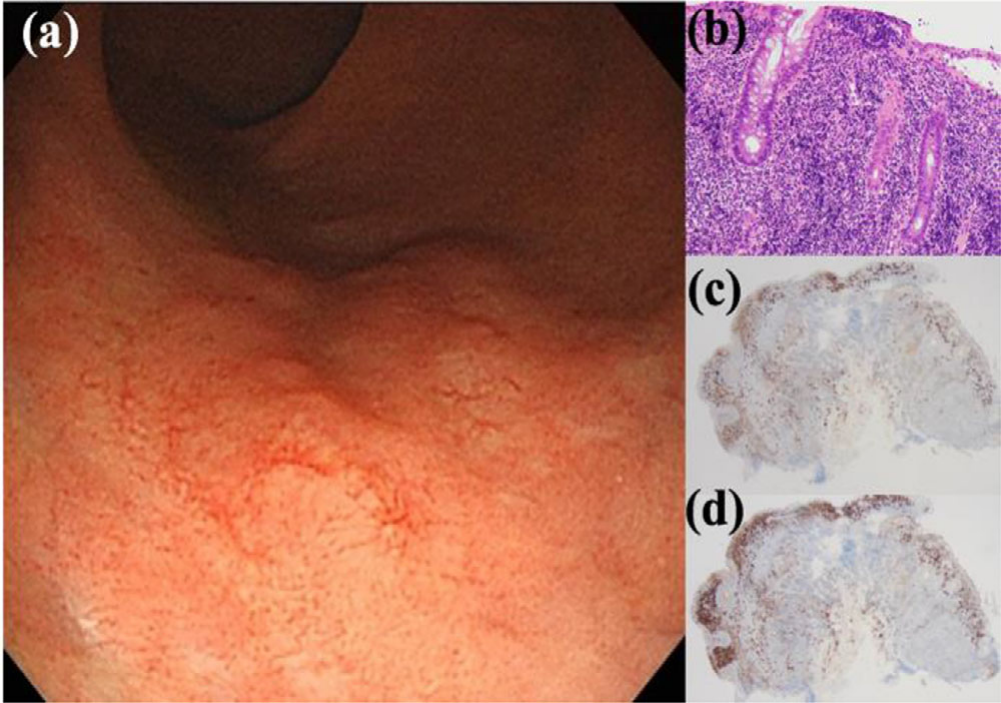
(a) The lesion spontaneously regressed 4 weeks after the first colonoscopy. (b) Examination of the biopsy specimen reveals an excessive infiltration of lymphocytes into the lamina propria without lymphoepithelial lesions. κ (c) and λ (d) immunohistochemical staining of the lymphoproliferative lesion. The κ/λ light‐chain restriction ratio is less than 2, indicating a benign lymphoid lesion

A 70‐year‐old man presented with pain during defecation with rectal bleeding. He had a history of myocardial infarction and was on oral clopidogrel. Rectal examination revealed a hard palpable mass that bled easily in the lower rectum. Colonoscopy revealed a large protruding reddish mass in the lower rectum, with mucosal erosion (Figure 1). SMTs such as malignant lymphoma, myogenic tumor, and neuroendocrine tumor were included in the differential diagnoses. Although there were inflammatory changes such as cryptitis and dense invasion of neutrophils into the mucosa, no malignant features were seen on forceps biopsy. Magnetic resonance imaging (MRI) also showed a 3.2 × 3.2 × 2 cm well‐defined mass on the anterior wall of the rectum, with homogeneous internal signal and no evidence of liver or lymph node metastasis (Figure 2). Contrast‐enhanced computed tomography (CT) showed a lower rectal mass with a contrast‐enhancing effect, suggesting the presence of a neoplasm. Due to the size and form of the lesion, endoscopic therapy could not be considered, and surgery was considered. Two weeks after the first biopsy, endoscopic ultrasonography (EUS) was performed, which showed that the lesion was a hypoechoic mass of the rectal wall, with absence of blood flow. Subsequent fine‐needle aspiration under EUS revealed many small to intermediate‐sized lymphocytes with no tumor components, although the size of the lesion had shrunk and symptoms, such as blood‐stained stool, tenesmus, and discomfort during defecation, had resolved. Since there was no evidence of malignancy, we decided to follow up on the case. Four weeks later, the protruded lesion appeared flat on colonoscopy (Figure 3), and a second forceps biopsy showed diffuse infiltrates of intermediate‐sized lymphocytes, suggesting a primary rectal lymphoma, especially mucosa‐associated lymphoid tissue lymphoma. Although CD3 and CD20 were positive on immunohistochemical staining, lymphoepithelial lesions were scarcely observed in the biopsy specimen (Figure 3). In addition, the ratio of κ and λ on immunostaining did not show light chain restriction; thus, there was no evidence of lymphoma monoclonality. Hence, the lesion was diagnosed as an RT.

**Figure 4:**
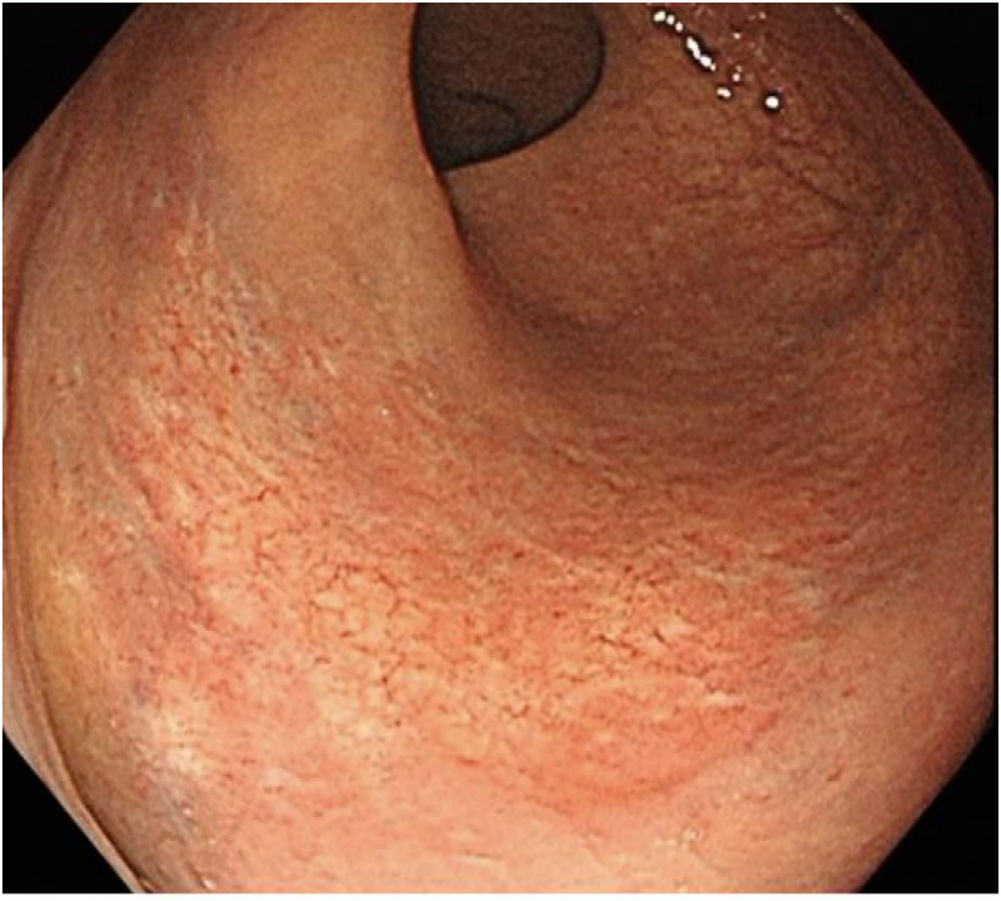
The lesion regresses with complete flattening and scarring

Four months after the initial colonoscopy, scar tissue had formed over the lesion (Figure 4), and 14 months after the diagnosis, the patient was asymptomatic. We plan to perform a routine endoscopic follow‐up.

## DISCUSSION

RTs, also known as benign lymphoid polyps, are localized lymphoid hyperplasia or benign lymphomas. They are caused by excessive diffuse and localized proliferation of lymphoid follicles in the rectal mucosa. The proliferation of lymphoid follicles is especially common in the terminal ileum but rarely occurs in the rectum. Therefore, it might be discovered incidentally; however, if the lesion is large, it might cause discomfort during defecation or hematochezia.^3^ RT is slightly more common in men and is seen across age groups ranging from infants to middle‐aged adults.^4^ There are a few reports about a viral or bacterial infection acting as a trigger for the onset; however, the exact mechanism remains unknown.^5^, ^6^

On endoscopy, the morphological form usually appears as a polypoid or serrated lesion with or without erosion; however, there are no specific features unique to RT. Hence, a diagnosis based on visual inspection is difficult, especially when differentiating between other types of SMTs such as malignant lymphoma, myogenic tumors, and neuroendocrine tumors.^2^ Another possible reason for the difficulty in making an endoscopic diagnosis of RT might be due to the rapid changes in its form caused by the acute inflammatory status, that is, lymphoid follicles proliferate when there is pathological inflammation, and when inflammation decreases, the lymphoid follicles soon regress. In this case, the patient had pain during defecation, and there was dense infiltration of neutrophils into the mucosa with cryptitis observed on histology after the first forceps biopsy. Hence, initially, there seemed to be presence of inflammation. Four weeks after the first forceps biopsy, the patients’ symptoms were alleviated, which indicates that the inflammation had also regressed. In addition to the submucosal changes, the active phase of inflammation is characterized by erosive changes and ulcer formation. As a reflection of these pathological changes, RTs could undergo significant morphological changes in a relatively short period.

Similar to ordinary endoscopy, EUS is reported to be a feasible modality for diagnosing rectal lesions and is superior to CT or MRI, especially for small lesions7. Differentiating between rectal tonsils, neuroendocrine neoplasms, and malignant lymphoma is difficult because all these lesions appear hypo to isoechoic on imaging and often originate from the second layer. Hence, procedures such as endoscopic resection are useful for both diagnosing and treating small lesions.^7^, ^8^ According to Cone et al.,^4^ RTs are often less than 1.5 cm in size, and such small lesions can be treated by endoscopic resection, and very few patients develop recurrence even with incomplete resection. On the other hand, relatively large lesions cannot be treated endoscopically, and diagnosis using biopsy specimens is even more difficult.^2^ Therefore, most of the reported cases were diagnosed using specimens excised endoscopically or surgically.

However, in general, less invasive treatment is preferred for neoplastic lesions of the rectum to preserve the function of the anal sphincter. Moreover, since RT is benign, it is important to be familiar with this disease to avoid misdiagnosis and overtreatment.

In this case, the patient was symptomatic, and surgical resection was initially considered. Fortunately, surgery could be circumvented due to the spontaneous shrinkage of the lesion and because it was diagnosed as RT due to repeated diagnostic endoscopies and biopsy. In retrospect, we should have performed a deeper biopsy, such as mucosal incision‐assisted biopsy, because a good biopsy specimen might have helped a definitive diagnosis at the first analysis.

However, from a clinical standpoint, our case is very valuable because it showed that even a large RT could regress spontaneously. Further, the natural course was followed until scar tissue developed over the lesion without excision. In retrospect, it can be deduced that repeated biopsies are useful for the diagnosis of RTs and for excluding other neoplasms. However, the natural course of RT remains unclear, and long‐term follow‐up is required.

In conclusion, RTs are rarely seen in the daily clinical practice of colonoscopy.

Clinicians should bear in mind that RTs are benign and self‐limiting entities, and the diagnosis should be made cautiously to avoid unnecessary surgery.

## CONFLICT OF INTEREST

The authors declare no conflicts of interest.

## FUNDING INFORMATION

None.
